# Roles of Dietary Bioactive Peptides in Redox Balance and Metabolic Disorders

**DOI:** 10.1155/2021/5582245

**Published:** 2021-06-09

**Authors:** Qinqin Qiao, Liang Chen, Xiang Li, Xiangyang Lu, Qingbiao Xu

**Affiliations:** ^1^College of Information Engineering, Fuyang Normal University, Fuyang 236041, China; ^2^College of Bioscience and Biotechnology, Hunan Agricultural University, Changsha 410128, China; ^3^College of Animal Sciences and Technology, Huazhong Agricultural University, Wuhan 430070, China

## Abstract

Bioactive peptides (BPs) are fragments of 2–15 amino acid residues with biological properties. Dietary BPs derived from milk, egg, fish, soybean, corn, rice, quinoa, wheat, oat, potato, common bean, spirulina, and mussel are reported to possess beneficial effects on redox balance and metabolic disorders (obesity, diabetes, hypertension, and inflammatory bowel diseases (IBD)). Peptide length, sequence, and composition significantly affected the bioactive properties of dietary BPs. Numerous studies have demonstrated that various dietary protein-derived BPs exhibited biological activities through the modulation of various molecular mechanisms and signaling pathways, including Kelch-like ECH-associated protein 1/nuclear factor erythroid 2-related factor 2/antioxidant response element *in oxidative stress*; peroxisome proliferator-activated-*γ*, CCAAT/enhancer-binding protein-*α*, and sterol regulatory element binding protein 1 in obesity; insulin receptor substrate-1/phosphatidylinositol 3-kinase/protein kinase B and AMP-activated protein kinase in diabetes; angiotensin-converting enzyme inhibition in hypertension; and mitogen-activated protein kinase and nuclear factor-kappa B in IBD. This review focuses on the action of molecular mechanisms of dietary BPs and provides novel insights in the maintenance of redox balance and metabolic diseases of human.

## 1. Introduction

Dietary food proteins contain short sequences of amino acids (AAs) that possess various biological activities. The short chains of AAs with biological properties are known as bioactive peptides (BPs). BPs usually contain 2–15 AA residues. The BP sequences present in the food proteins are generally hidden in the inner core of the parent proteins [1]. However, bioactive peptides can be released from food proteins with the help of several techniques including proteolysis, microbial fermentation, and gastrointestinal (GI) digestion [2–4]. Among these methods, in vitro enzymatic hydrolysis using commercial proteolytic enzymes is the widely employed method in recent times. The proteases cleave the specific sites on the dietary proteins and release the short chains of BPs [5]. Enzymatic hydrolysis of dietary proteins enhances the nutritional value, bioactivity, and functionality and reduces the allergenicity [6]. Several commercial proteases, namely, thermolysin, bromelain, trypsin, alcalase, papain, pepsin, neutrase, pancreatin, corolase, protamex, and pronase, have been employed to generate biologically active peptides from various dietary proteins. Among the above-mentioned proteases, alcalase, trypsin, pepsin, and pancreatin are the most widely used proteases for the preparation of peptides, with various health benefitting properties, from numerous food sources [7–10]. These enzymes are routinely used due to their broad specificity to produce smaller peptides with various bioactivities and easily availability.

Dietary BPs have been shown to positively affect the various systems of the human body including the immune, cardiovascular, GI, and nervous systems [11]. The BPs must cross the GI barrier and reach the target tissue or organ in order to exhibit health benefits. Dietary peptides exert bioactivities through the modulation (either enhance or decrease) of various molecular mechanisms and pathways. Bioactive properties of dietary peptides are affected by the peptide sequence, length, hydrophobicity, and composition [12–14]. Dietary peptides are easily absorbable across the intestinal border via peptide transport 1 (PepT1) and possess excellent functional properties (solubility, foaming, and emulsification properties) [15–18]. Additionally, dietary BPs are generally safer than synthetic drugs. Hence, BPs obtained from dietary sources could be used as health foods/nutraceuticals in the management/prevention of various diseases. Numerous BPs derived from whey, casein, milk, soybean, shark, bonita, pacific whiting, porcine, and bovine have been on the market in various countries for human use as functional foods/health foods/nutraceuticals [19].

**Figure 1:**
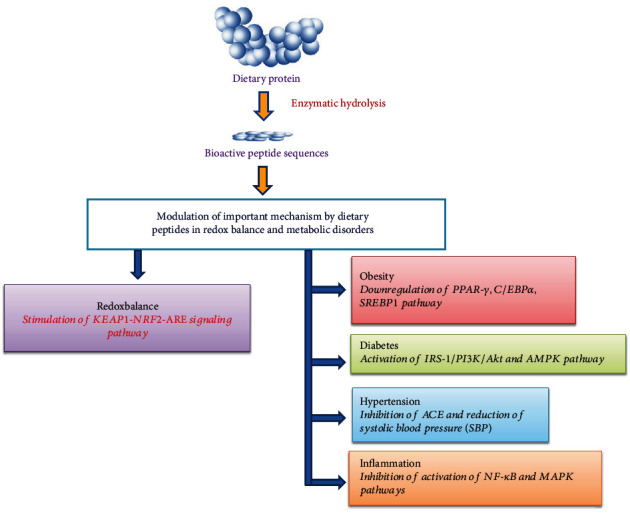
Effects of dietary bioactive peptides on molecular mechanisms involved in redox balance and metabolic disorders. ACE: angiotensin-converting enzyme; Akt: protein kinase B; AMPK: AMP-activated protein kinase; ARE: antioxidant response element; C/EBP: CCAAT-enhancer-binding proteins; IRS-1: insulin receptor substrate; PPAR: peroxisome proliferator-activated receptor; Keap1: Kelch-like ECH-associated protein 1; MAPK: mitogen-activated protein kinase; NF*-κ*B: nuclear factor-*κ*B; *Nrf2*: nuclear factor erythroid 2-related factor 2; PI3K: phosphatidylinositol 3-kinase; SREBP1: sterol regulatory element-binding protein 1.

Redox homeostasis (balance) is an important cellular process that plays a vital role in the maintenance of a normal physiological steady state [20]. Disturbance of balance between oxidants and antioxidants results in the oxidative stress. Recently, many BPs prepared from various food sources such as walnut, egg, fish, quinoa, soybean, millets, corn, wheat, rice, potato, milk, and spirulina have shown to possess beneficial effects in the maintenance of redox homeostasis and prevention/management of metabolic diseases [21–26]. However, reviews describing the role of dietary BPs in the modulation of molecular mechanisms of redox balance and metabolic diseases are scanty in the literature. Hence, the current review focuses on the recent literature related to the effects of dietary BPs on various molecular mechanisms and signaling pathways involved in the redox homeostasis and metabolic diseases (obesity, diabetes, hypertension, and inflammation), as shown in Figure 1.

## 2. Roles of Dietary Bioactive Peptides in Maintaining Redox Balance

Redox reaction is a chemical reaction that involves the transfer of electrons from the reducing agent to the oxidizing agent [27]. Redox homeostasis (balance) plays a significant role in the maintenance of ordinary physiological functions of the human body. Redox balance is considerably affected by reactive oxygen species (ROS) that are generated during aerobic cellular metabolism [28]. Reactive oxygen species are unstable and highly reactive in the redox reactions due to the presence of unpaired electrons in the outer shells. Under normal physiological conditions, redox balance is maintained through careful regulation of ROS generation and elimination from the body [29]. However, excess production of ROS during oxidative stress conditions could alter the intracellular redox balance and promote the development of numerous diseases such as cancer, diabetes, atherosclerosis, cardiovascular, and neurodegenerative diseases. The body's natural antioxidant defense system, namely, superoxide dismutase (SOD), glutathione peroxidase (GPx), and catalase (CAT), plays a vital role in the maintenance of balance between ROS formation and elimination [30].

Several studies have demonstrated that a number of peptides identified from various dietary sources have shown the ability to suppress oxidative stress and maintain the redox balance through multiple molecular mechanisms such as scavenging free radicals; chelating transition metals; enhancing the production of endogenous antioxidant enzymes SOD, CAT, and GPx; and stimulating the nuclear factor erythroid 2-related factor 2 (Nrf2) antioxidant defense mechanism [12, 15, 17, 26, 31–34]. The antioxidant mechanisms showed by the dietary peptides mainly depend on the peptide sequence, length, composition, and hydrophobicity [12, 34, 35]. The food-derived antioxidant peptides usually contain 3–15 AA residues [12, 32].  Table 1 shows the AA sequences and molecular mechanisms of antioxidant peptides produced from various dietary proteins.

The Kelch-like ECH-associated protein 1- (*Keap1*-) *Nrf2*-antioxidant response element (ARE) is the main antioxidant signaling pathway that prevents oxidative stress and helps maintain the optimum redox steady state in the body [36]. The *Nrf2* is a vital leucine zipper transcription factor, which controls the expression of several antioxidant proteins in response to ROS stress. Keap1 is a suppressor protein for Nrf2, and under a normal ROS steady state, Keap1 binds with Nrf2 and helps proteasome to degrade the Keap1-Nrf2 complex. However, Nrf2 separates from Keap1 during oxidative stress and migrates to the nucleus where it attaches to ARE and thereby promotes the expression of several antioxidant enzymes/proteins [32, 37, 38]. Endogenous antioxidant enzymes such as SOD, GPx, and CAT scavenge different kinds of ROS and thereby protect the cells from oxidative stress-induced damage. SOD catalyses the transformation of superoxide anion to O_2_ and H_2_O_2_. CAT converts H_2_O_2_ to H_2_O and O_2_. GPx helps in the reduction of H_2_O_2_ to H_2_O and O_2_ [39]. Hence, it is important to stimulate the Nrf2 antioxidant signaling pathway to suppress/prevent the oxidative stress in the body.

Recently, numerous novel antioxidant peptides that stimulate the *Keap1*-*Nrf2*-ARE *antioxidant signaling pathway and antioxidant enzymes* have been isolated from different dietary sources such as casein [40], milk protein concentrate [41], corn gluten [31], soybean [42], walnut [43], potato, *Moringa oleifera* seeds [25], watermelon seeds [39], *Crassostrea rivularis* [44], krill [38], turtle [45], *Mytilus coruscus* [46], *Channa argus* [3], and silver carp muscle [12]. Snakehead (*Channa argus*) soup was hydrolyzed using pepsin and pancreatin and identified four antioxidant peptides, IVLPDEGK, PGMLGGSPPGLLGGSPP, SDGSNIHFPN, and SVSIRADGGEGEVTVFT [3]. The authors performed molecular docking studies for the peptides and indicated that peptides could bind to the active site of Keap1 and thereby activate the cellular antioxidant Keap1-Nrf2 signaling pathway. A peptide RDPEER isolated from alcalase hydrolysate of watermelon seed reduced the oxidative stress by reducing ROS and increasing the antioxidant enzymes, SOD, CAT, and GSH-Px, in HepG2 cells [39]. Four casein-derived peptides, ARHPHPHLSFM, AVPYPQR, NPYVPR, and KVLPVPEK, were shown to decrease the oxidative stress in Caco-2 cells by enhancing antioxidant enzymes, namely, SOD1, Trx1, GR, TrxR1, and NQO1, through the activation of the Keap1-Nrf2 signaling mechanism. It was found that the peptides bound to the Nrf2 and prevented the binding between Keap1 and Nrf2 and thereby stimulated the increased expression of antioxidant enzymes [32]. In a study, a peptide, AMVDAIAR, isolated from pepsin hydrolysate of krill enhanced antioxidant enzymes SOD, CAT, and GPx and thereby suppressed the oxidative stress in H_2_O_2_-induced hepatocytes through increasing the expression of Nrf2 [38]. Peptide EDYGA derived from soft-shelled turtle enhanced the Nrf2 level through the binding of the glutamate residue of the peptide to Arg 415 of the Kelch receptor pocket [45]. Dipeptide IF identified from potato exhibited antioxidant effects by increasing the antioxidant enzymes GPx 4, SOD1, HO-1, SOD2, and peroxiredoxin 2 in the kidney tissues of the spontaneously hypertensive rats (SHRs) [33]. The authors concluded that the dipeptide showed antioxidative activity through preventing Nrf2 degradation by protein kinase B (Akt) activation and GSK-3*β* phosphorylation.

## 3. Role of Dietary Bioactive Peptides in Obesity

Obesity is a public health issue worldwide and characterized by excessive body fat accumulation due to the difference between energy intake and energy spending, which enhances the risk of metabolic disorders, namely, type 2 diabetes mellitus (T2DM), hypertension, and cardiovascular disorders [47, 48]. It is estimated that globally, 18% men and 21% women will suffer from obesity by 2025 [49]. Adipocytes are fat cells and the main function of adipocytes is the storage of energy as fat. Preadipocytes differentiate into mature adipocytes through a process known as adipogenesis. Hypertrophy and hyperplasia of adipocytes are two mechanisms that contribute to the obesity and obesity-related metabolic disorders [50, 51].

Several recent studies demonstrated that various dietary peptides from soybean, quinoa, common bean, camel whey, spirulina, blue mussel, skate, tuna, Alaska pollock, sardinella, hazelnut, and kefir exhibited antiobesity effects through modulation of multiple molecular mechanisms including the reduction of adipogenesis through downregulation of the expression of peroxisome proliferator-activated receptor- (PPAR-) *γ*, CCAAT/enhancer binding protein alpha (C/EBP-*α*), sterol regulatory element binding protein- (SREBP-) 1, and HMGCR, enhancing lipolysis, reducing body weight (BW) and food intake, inhibiting lipase activity, decreasing the accumulation of triglycerides, and blocking lipogenesis by reducing the fatty acid synthase [2, 10, 52, 53]. Various molecular mechanisms of antiobesity peptides are shown in Table 2.

During maturation of preadipocytes into adipocytes, several transcriptional factors are involved. PPAR and C/EBP are two transcription factors that stimulate adipocyte differentiation [50]. Lipid and carbohydrate metabolism is regulated by the PPARs. SREBP1 is a lipogenic transcription factor upon activation by PPAR-*γ* which promotes the adipogenesis and lipogenesis. SREBP1 stimulates lipoprotein lipase and fatty acid synthase and thereby enhances the lipid accumulation in the adipocytes [50]. Therefore, inhibition of the PPARs, C/EBP, and SREBP1 transcriptional factors involved the adipogenesis and lipogenesis using dietary-derived BPs is an efficient approach in the prevention or treatment of obesity and related diseases. It has been revealed that numerous dietary peptides from soy bean, quinoa, hazelnut, canola, tuna, ark shell, and blue mussel showed antiobesity activities by inhibiting the expression of PPAR-*γ*, C/EBP, and SREBP1 transcriptional factors [10, 51, 54].

It was found that the antiobesity effects of dietary BPs are related to the peptide sequence, length, composition, and protein source [13]. Peptides with < 1 kDa from blue mussel by pepsin hydrolysis exhibited antiobesity effects by enhancing lipolysis and downregulating adipogenic transcription factors, such as PPAR-*γ*, C/EBP-*α*, and SREBP1 [10]. An antiobesity pentapeptide RLLPH was isolated from alcalase hydrolysate of hazelnut (*Corylus heterophylla* Fisch) and found that the pentapeptide could decrease adipogenesis through reducing the expression of PPAR-*γ*, C/EBP-*α*, SREBP-1c, adipocyte protein (aP2), FAS, acetyl-CoA carboxylase 1 (ACC1), and HMGCR in 3T3-L1 adipocytes [13]. Additionally, the authors indicated that the hydrophobic AAs, proline, and leucine, of the peptide, might have contributed to the antiobesity effects of the peptides. Peptides with more hydrophobic AAs can easily penetrate the cell membrane and increase lipid solubility. In a study, peptides < 1 kDa from ark shell (*Scapharca subcrenata*) protein inhibited intracellular lipid buildup and enhanced the lipolysis [51]. The authors were also demonstrated that ark shell peptides inhibited adipogenesis by decreasing the expressions of PPAR-*γ*, C/EBP-*α*, SREBP-1c, lipoprotein lipase, and FAS in mouse mesenchymal stem cells. Moreover, the expression of PPAR-*γ*, C/EBP-*α*, and aP2 was decreased by tuna skin collagen-derived peptides in obese mice, which resulted in the decrease of adipocyte size [55].

In addition to the downregulation of vital transcriptional factors (PPAR-*γ*, C/EBP-*α*, and SREBP-1c) of adipogenesis, several in vivo studies demonstrated that the antiobesity activity of dietary peptides is due to the decrease of BW and food consumption. Oral administration of peptides (10 mg/mL), produced from smooth hound (*Mustelus mustelus*) muscle by alkaline crude enzyme from *M. mustelus* intestines, for 21 days reduced the BW and food intake in rats [5]. The authors suggested that BW reduction was probably due to the regulation of appetite. The antiobesity effect of Alaska pollack-derived peptides was investigated, and it was found that peptide administration (100 or 300 mg/kg BW) to rats for 3 days decreased the weight of white adipose tissue and reduced the food intake [56]. The authors suggested that the decrease in food intake and white adipose tissue weight of rats after peptide treatment was due to downregulation/suppression of gene expressions of *neuropeptide-Y* and agouti-related peptide in hypothalamus, which may reduce the appetite. In a study, it was found that antiobesity effects of sardinella- (*Sardinella aurita*-) derived peptides were mediated by reducing the BW gain, food intake, and the relative epididymal adipose tissue weight in Wistar rats after 10 weeks of peptide treatment [2]. It was demonstrated that *Spirulina platensis*-derived peptides exhibited antiobesity effects by reducing BW (39.8%) and lowering serum glucose (23.8%) through altering the gene expressions of Acadm, Retn, Fabp4, Ppard, and Slc27a1 in the brain and liver of mice fed with peptides (2 g/kg BW) for 4 weeks [57]. Recently, it was reported that walleye pollock skin collagen-derived peptides considerably reduced the BW gain in obese mice after 8 weeks of peptide treatment [58]. The authors also pointed that peptides inhibited the growth of adipocytes and the accumulation of adipose tissue in obese mice. Furthermore, in a placebo-controlled, randomized clinical investigation, salmon fish-derived peptide supplementation (16 g/d) for 42 days notably decreased (5.6%) the BMI in obese humans [59].

Pancreatic lipase is an important enzyme that aids in the hydrolysis of dietary fat in the small intestine. Therefore, inhibition of pancreatic lipase is an efficient approach in the management and treatment of being overweight and obesity [2, 24]. Apart from inhibition of adipogenesis and lipogenesis, some dietary peptides exhibited pancreatic lipase inhibitory activity. Peptides produced from camel milk by using alcalase-, bromelin-, and papain-inhibited porcine pancreatic lipase [24]. It was also found that sardinella peptide administration to rats for 10 weeks decreased the pancreatic lipase activity [2].

## 4. The Role of Dietary Bioactive Peptides in Diabetes Mellitus

Diabetes mellitus (DM) is a complex metabolic disorder with increased blood sugar levels and T2DM accounts for 90% of diabetes patients. T2DM results from insulin resistance (cells less responsive to the insulin actions) and/or insufficient insulin production from pancreatic beta cells. Obesity (BMI ≥ 30 kg/m^2^) has been reported to greatly increase the risk of developing T2DM [60]. Uncontrolled T2DM can cause severe complications such as high blood pressure, stroke, heart attack, atherosclerosis, retinopathy, nephropathy, neuropathy, and dementia.

Recently, several peptides derived from a variety of dietary protein sources including egg white, whey, casein, egg yolk, rice bran, quinoa, soybean, wheat, corn, black bean, oat globulin, walnut, potato, common bean, millets, spirulina, bovine, porcine, Atlantic cod, Atlantic salmon, halibut skin, *Styela clava*, boarfish, tilapia skin, largemouth bass, zebra blenny, blue whiting, and sea cucumber have shown antidiabetic effects by altering several molecular mechanisms of diabetes such as inhibition of enzymes including *α*-amylase, dipeptidyl peptidase- (DPP-) IV, and *α*-glucosidase; reduction of FBG and HbA1C; enhancement of HOMA-IR; stimulation of secretion of glucagon-like polypeptide-1 (GLP-1) and insulin levels; upregulation of phosphatidylinositol 3-kinase (PI3K), p-GSK-3*β*, p-Akt, and glucose transporter (GLUT)2/4 signaling pathways; blocking of glucose transporters GLUT2 and SGLT1; decreasing of the activation of p38 and c-Jun N-terminal kinase (JNK)1/2; enhancement of the stimulation of insulin receptor substrate-1 (IRS-1) tyrosine residue and Akt; and decreasing of gluconeogenesis through activation of IRS-1/PI3K/Akt and AMP-activated protein kinase (AMPK) [6, 21, 61–64]. The isolated peptide sequences and molecular mechanisms of dietary antidiabetic peptides are shown in Table 3.

*α*-Amylase is an important enzyme in the carbohydrate digestion that breaks down *α*-1,4 glycosidic linkages of starch and produces oligosaccharides. *α*-Glucosidase is present in the brush borders of the small intestine and hydrolyzes the disaccharides and starch to glucose by acting upon *α*(1 → 4) glycosidic bonds. Therefore, inhibition of *α*-amylase and *α*-glucosidase prevents carbohydrate digestion and thus diminishes the postprandial increase of blood glucose. Several chemical *α*-glucosidase and *α*-amylase inhibitors (acarbose, voglibose, and miglitol) have been in use for the management and treatment of T2DM [65]. However, side effects associated with these inhibitors limited their use [63]. Recently, many peptides isolated from several food sources including egg white, corn, oat, egg yolk, spirulina, quinoa, soybean, *Phaseolus vulgaris*, and zebra blenny have exhibited *α*-amylase and *α*-glucosidase inhibitory activities [61, 62, 66, 67]. Egg white albumin was hydrolyzed using alcalase, and a pentapeptide KLPGF was isolated with *α*-glucosidase (50% inhibitory concentration (IC_50_) 59.5 *μ*mol/L) and *α*-amylase (IC_50_ 120 *μ*M) inhibitory activities [66]. Three peptides, GVPMPNK, LRSELAAWSR, and RNPFVFAPTLLTVAAR, were extracted from spirulina platensis, and it was found that peptide LRSELAAWSR strongly inhibited *α*-amylase with IC_50_ of 313.6 *μ*g/mL and *α*-glucosidase with IC_50_ of 134.2 *μ*g/mL [68]. A *α*-glucosidase inhibitory peptide, LAPSLPGKPKPD, was identified from egg yolk hydrolysate produced by proteinase from Asian pumpkin with an IC_50_ value of 1065.6 *μ*mol/L [67]. Three peptides, LLPLPVL, SWLRL, and WLRL, produced from soy protein showed *α*-glucosidase inhibitory activity with IC_50_ 162.2–237.4 *μ*mol/L [63]. A study reported isolation of *α*-glucosidase inhibitory peptide, QHPHGLGALCAAPPST, from quinoa with an IC_50_ of 1.0–1.45 mg/mL [62]. Recently, corn germ protein was hydrolyzed by using alcalase, trypsin, and flavourzyme and it was found that the peptide fraction (2–10 kDa) showed strong *α*-amylase inhibition (71.3%) and *α*-glucosidase inhibition (37.1%) activities [61].

Another strategy for the management and treatment of T2DM is to inhibit the DPP-IV that degrades and inactivates incretin hormones, namely, glucose-dependent insulinotropic peptide (GIP) and GLP-1. Therefore, inhibition of DPP-IV by dietary-derived BPs can enhance the half-life of GLP-1 and thereby increase the release of glucose-dependent insulin from pancreatic cells [61]. For inhibition of DPP-IV activity, several synthetic drugs such as saxagliptin, linagliptin, sitagliptin, and vildagliptin are presently used. However, side effects (diarrhea, nausea, stomach pain, headache, and sore throat) associated with these drugs have forced researches to search for DPP-IV inhibitors from natural dietary sources without any side effects [69]. Many recent studies have reported that dietary protein-derived peptides are an excellent source of DPP-IV inhibitors. Peptides obtained from millet, corn, quinoa, egg yolk, oat, whey, casein, *Spirulina platensis*, porcine, Atlantic salmon, sea cucumber, largemouth bass, blue whiting, and *Capros aper* inhibited the DPP-IV activity [67, 70–72]. It has been found that dietary peptides inhibit DPP-IV through attaching the peptides to the active sites of DPP-IV via hydrogen bonds and hydrophobic interactions and thereby prevent the enzyme action [14, 21]. Two peptides NDWHTGPLS and TYPHQQPPILT derived from papain hydrolysates of millet proteins inhibited DPP-IV activity (75%) [21]. It was demonstrated that both the peptides inhibited DPP-IV activity by occupying the active sites of DPP-IV via hydrogen and pi bonds. In a recent study, Atlantic salmon skin was hydrolyzed by using trypsin and isolated a new DPP-IV inhibitory peptide, LDKVFR, with IC_50_ of 128.7 *μ*M [14]. Additionally, it was found that 6 H bonds and 8 hydrophobic interactions played a significant role in the inhibition of DPP-IV by LDKVFR. A peptide, LDQWLCEKL, obtained from trypsin hydrolysate of *α*-lactalbumin-rich whey proteins inhibited DPP-IV (IC_50_ 131 *μ*M) through a noncompetitive mode of inhibition.

The PI3K/Akt signaling pathway regulates the glucose uptake. When cells are resistant to insulin, glucose uptake is impaired in the liver and skeletal muscles. Insulin activates IRS after binding to IRS and the activated IRS phosphorylates IRS-1. Phosphorylation of IRS-1 results in the activation of PI3K. The activated PI3K phosphorylates Akt, and the activated Akt helps in the migration of intracellular GLUT2/4 to the plasma membrane and therefore increases the glucose absorption into cells. But insulin resistance weakens the PI3K/Akt signaling pathway [73, 74]. Hence, stimulation of the PI3K/Akt molecular pathway is an efficient approach in the management of insulin resistance. In a study, the authors hydrolyzed walnut protein using alcalase and isolated an antidiabetic peptide LPLLR with a molecular weight of 610.4 Da. The authors reported that the identified peptide improved hepatic insulin resistance (IR) through enhancing glycogen synthesis and glucose uptake and reducing gluconeogenesis via activating the IRS-1/PI3K/Akt and AMPK signaling pathways in hepatic HepG2 cells [75]. Two hundred forty-two peptides, with a molecular weight ranging from 203 to 1907 Da, were isolated from the hydrolysate of sea cucumber and found that the peptides showed antidiabetic effects by upregulation of PI3K, p-Akt, p-GSK-3*β*, and GLUT2/4 signaling pathways, while decreasing p-IRS1 expression in diabetic rats [73].

In addition to the peptides described above, the antidiabetic activities of dietary peptides have also been investigated in human subjects. In a randomized and crossover clinical trial, whey peptide (<5000 Da) (1400 or 2800 mg/kg BW) administration to 21 prediabetic human subjects decreased under the curve (iAUC) of glucose as well as showed a minor insulinotropic effect and reduced HbA1c [76]. A double-blind crossover clinical trial conducted using peptides (<2000 Da) derived from Atlantic cod showed that a single dose of 20 mg/kg BW considerably decreased the postprandial insulin in 41 healthy individuals [77]. Moreover, peptides derived from *Styela clava* have also been shown to decrease the hemoglobin A1c and plasma insulin levels after 4 weeks of administration in patients with T2DM [78].

## 5. The Role of Dietary Bioactive Peptides in Hypertension

Hypertension is an important risk factor that can increase the chance of developing heart attack or stroke. Clinically, systolic blood pressure (SBP) 140 mmHg or above and/or DBP 90 mmHg or above are considered as hypertension [79]. It is estimated that over a billion people (1 in 5 women and 1 in 4 men) are suffering from hypertension.

Food-derived peptides play a significant role in the prevention of hypertension. Recently, numerous antihypertensive peptides are isolated from different food sources such as milk [80], casein [81, 82], egg white ovotransferrin [22], rice bran [1], wheat [83], soybean [84], potato [85], turmeric and ginger [9], quinoa [62], black cumin [86], coix [87], pistachio [88], hazelnut [89], mung bean [90], lentil [91], seahorse [92], egg white from ostrich [93], chum salmon [94], skate [95], cuttlefish [96], Sipuncula [97], bighead carp [98], shrimp (*Pandalus borealis*) [79], and beef [99]. The isolated dietary protein-derived peptides have been demonstrated to exhibit antihypertensive activities through influencing various molecular mechanisms including inhibition of angiotensin-converting enzyme (ACE), reduction of SBP, decrease of angiotensin II levels and AT1R expression, enhancing vasodilation, improving central blood pressure and arterial stiffness, and inhibition of vasoconstriction via PPAR-*γ* expression [9, 79, 84, 90].  Table 4 shows the molecular mechanisms of antihypertensive peptides isolated from various dietary sources.

Human blood pressure is regulated by ACE (EC 3.4.15.1). ACE cleaves the dipeptide, HL, from angiotensin I and converts the inactive angiotensin I into angiotensin II and thereby enhances the blood pressure. Angiotensin II is a powerful vasoconstrictor, and ACE inactivates the bradykinin, which is a potent vasodilator. Therefore, inhibition of ACE is an important molecular target in the prevention and management of hypertension. Currently, several peptide drugs, namely, captopril, lisinopril, and enalapril, are used as ACE inhibitors for the management of high blood pressure [80, 97, 100]. Due to the side effects (cough, fatigue, dizziness, headaches, and loss of taste) associated with these synthetic drugs, there is an increasing interest to search for safe ACE inhibitors from natural food sources. Various food sources are an excellent source of ACE inhibitory peptides. Most dietary ACE inhibitory peptides contained 3–15 AA residues. Recently, several peptides have been isolated with ACE inhibitory activity from numerous dietary sources such as milk [80], casein [82], egg white [93], soybean [84], rice bran [1], wheat [83], pistachio [88], potato and rapeseed [85], turmeric and ginger [9], quinoa [62], black cumin [86], hazelnut [89], lentil [91], seahorse [92], chum salmon [94], skate [95], cuttlefish [96], Sipuncula [97], bighead carp [98], and beef [99].

It has been demonstrated that hydrogen bonds play a vital role in the binding of BPs to the ACE catalytic pocket and thereby facilitate ACE inhibition. Three ACE inhibitory peptides, LLSGTQNQPSFLSGF, NSLTLPILRYL, and TLEPNSVFLPVLLH, were isolated from lentil seeds with IC_50_ of 44–120 *μ*M, and it was found that the peptides inhibited ACE through interactions by hydrogen bonds with three residues of the ACE catalytic site [91]. A tripeptide YSK with ACE inhibition was identified from trypsin hydrolysate of rice bran, and ACE inhibition of YSK was due to the formation of hydrogen bonds with the binding site of ACE [1]. The molecular interactions between dipeptide, YV, obtained from ostrich egg white and ACE were studied and it demonstrated that YV inhibited ACE (IC_50_ 63.97 *μ*g/mL) by binding to S1 and S2 pocket sites of ACE through hydrogen bonds [93]. A pentapeptide, ACKEP, purified from pistachio kernel hydrolysates was shown to inhibit ACE (IC_50_ 126 *μ*M) by binding with seven AAs of the ACE catalytic site (His383, His387, Glu384, Arg522, Asp358, Ala356, and Asn70) and two atoms of ACKEP [88]. In a study, three ACE inhibitory peptides, AVKVL, YLVR, and TLVGR, were identified from alcalase hydrolysate of hazelnut. The authors found that all the three peptides exhibited ACE inhibition (IC_50_ 5.42–249.3 *μ*M) via a noncompetitive inhibition through the formation of cation–pi interactions [89]. In another study, four ACE inhibitory peptides, EDEVSFSP, SRPFNL, RSPFNL, and ENPFNL, were purified from fermented soybean with IC_50_ of 0.131–0.811 mg/mL [84]. It was suggested that the N-terminal sequence and the location of AAs in the peptides play an essential role in ACE inhibition. Recently, a peptide, VTPVGVPK, produced by *α*-chymotrypsin hydrolysis from black cumin seed was shown to inhibit ACE (IC_50_ 1.8 *μ*M) through a noncompetitive inhibition [86]. A peptide, QHPHGLGALCAAPPST, identified from chymotrypsin hydrolysate of quinoa inhibited ACE by binding to the number of active hotspots of the ACE enzyme [62]. Moreover, two peptides, SAGGYIW and APATPSFW, were isolated from wheat gluten with ACE inhibition of IC_50_ 0.002–0.036 mg/mL [83]. The authors concluded that two peptides with proline and negatively charged residues inhibited ACE through the modulation of ionic and hydrophobic connections of the ACE active site.

In addition to ACE inhibition, several in vivo studies (animal and human) have reported the blood pressure-lowering effects of numerous peptides isolated from various food sources. Peptides, SLVSPSAAAAAAPGGS and KKRSKKKSFG, generated from potato and rapeseed were found to reduce (154.7 mmHg) the mean arterial blood pressure of treated rats compared to the control (177 mmHg) group [85]. Two antihypertensive peptides, IQW and LKP, were identified from thermolysin and pepsin hydrolysates prepared from egg white ovotransferrin and demonstrated that tripeptide (IQW and LKP) administration reduced the mean blood pressure by 19 and 30 mmHg, respectively, compared to control SHRs [22]. Administration of a peptide, VELYP, produced from *Sepia officinalis* muscle, to SHR exhibited antihypertensive effects by decreasing SBP [96]. In a study, a beef myofibrillar protein-derived peptide LIVGIIRCV at 400 and 800 mg/kg BW decreased SBP by 28 and 35 mmHg in SHRs, respectively [99]. A randomized and double-blind human trial conducted on level 1 hypertensive patients demonstrated that eight-week supplementation of casein-derived tripeptides, VPP and IPP, ameliorated central blood pressure and arterial stiffness [81]. In a recent randomized, double-blind clinical trial, the administration of shrimp-derived peptides (1200 mg/d) for eight weeks reduced the BP in mild- or moderate-hypertension patients [79]. Additionally, the authors suggested that the reduction of BP was probably due to the decrease of angiotensin II levels in hypertension patients.

## 6. The Role of Dietary Bioactive Peptides in Inflammatory Bowel Diseases (IBD)

Inflammation is a complex and natural response of the body in an attempt to resolve harmful stimuli such as pathogens, tissue injuries, infections, or toxins. However, uncontrolled and chronic inflammation has been reported to be linked with several diseases such as T2DM, metabolic syndrome, IBD, cardiovascular disease, cancer, asthma, arthritis, and chronic obstructive lung disease [101]. IBD symptoms such as diarrhea, abdominal pain, fever, vomiting, BW loss, and rectal bleeding affect the quality of life of patients [102]. Crohn's disease (DC) and ulcerative colitis (UC) are two major forms of IBD [103]. UC is the inflammation of colon. Chronic inflammation in the intestine produces excessive and uncontrolled proinflammatory cytokines including tumor necrosis factor-alpha (TNF-*α*), interleukin- (IL-) 1*β*, IL-6, IL-8, IL-12, interferon-gamma (IFN-*γ*), and IL-17 [102, 103].

**Figure 2:**
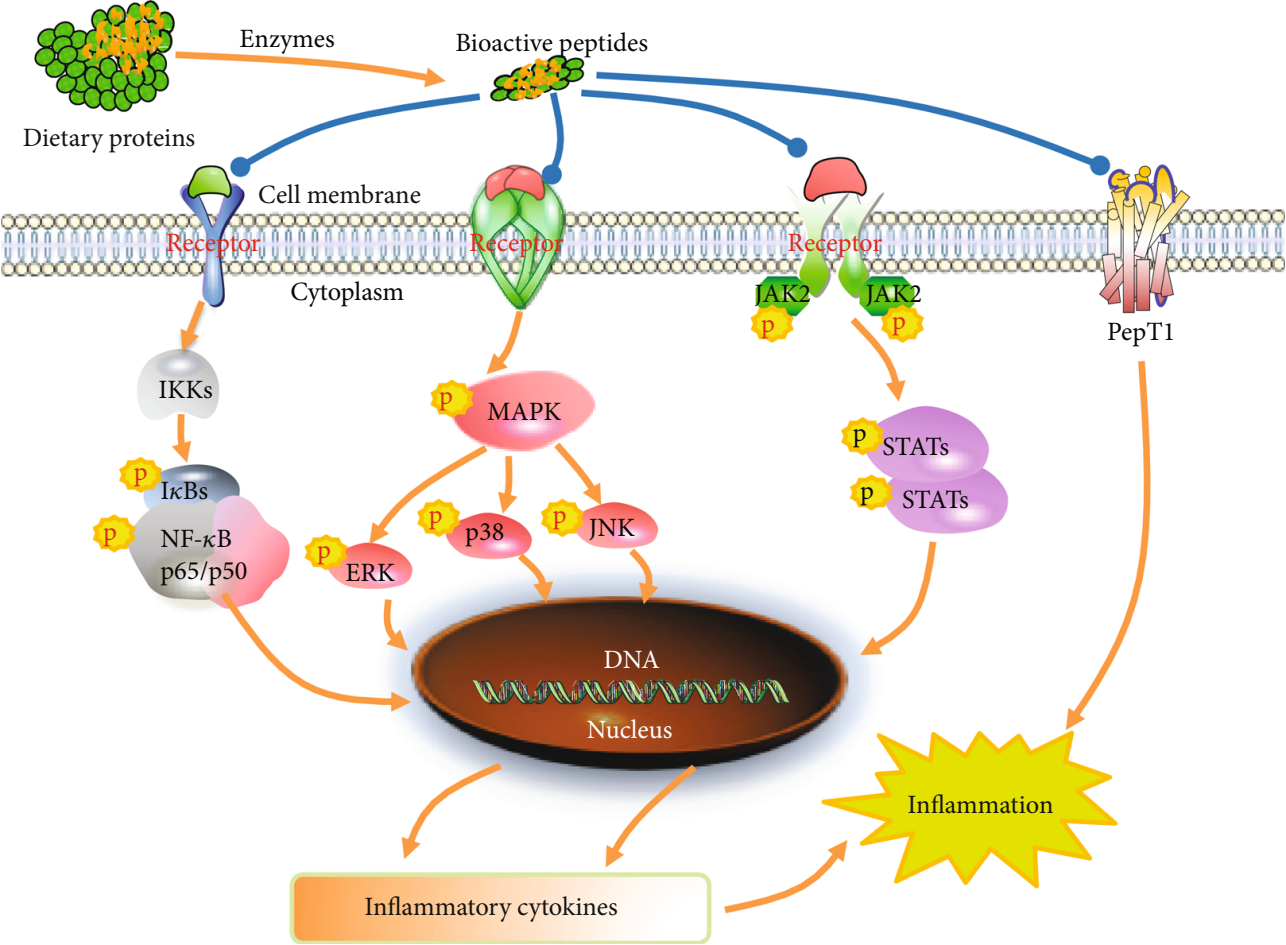
The mechanism of anti-inflammation of dietary protein-derived bioactive peptides: NF-*κ*B, MAPK, JAK2-STAT, and PepT1. ERK: extracellular signal-regulated kinases; MAPK: mitogen-activated protein kinase; NF-*κ*B: nuclear factor-kappa B; JAK-STAT: Janus kinase-signal transducer and activator of transcription; JNK: c-Jun N-terminal kinase; p: phosphorylation; PepT1: peptide transport 1.

The mechanism of anti-inflammation by food protein-derived BPs is that they can inhibit the phosphorylation of signaling pathways including nuclear factor-kappa B (NF-*κ*B), mitogen-activated protein kinase (MAPK), Janus kinase-signal transducer and activator of transcription (JAK-STAT), and peptide transporter PepT1 as shown in Figure 2 [26, 104, 105]. The NF-*κ*B pathway contains IKKs, I*κ*Bs, and p65/p50, while the MAPK pathway contains p38, JNK, and extracellular signal-regulated kinases (ERK). BPs can inhibit the NF-*κ*B receptor, resulting in the inhibition of the activation of inhibitory *κ*B kinases (IKK*α*/*β*/*γ*), which can lead to phosphorylation of cytoplasmic transcription factor (I*κ*B*α*/*β*/*γ*) and I*κ*B*α* degradation. BPs can inhibit the MAPK receptor and inhibit MAP3K phosphorylation, which can mediate the phosphorylation of the downstream MAP2K and MAPK. The inhibition of phosphorylation of MAPK and JAK2-STATs by BPs can alleviate the release of cytokines. The BP inhibition of translocations of the above transcription factors in nucleus (ATF-2, AP-1, and c-Jun) can cause the gene change, reducing the productions of proinflammatory cytokines, such as IL-1*β*, IL-6, IL-8, TNF-*α*, and IFN-*γ*, resulting in the inflammation suppression (Figure 2). In addition, the PepT1 can transport small BPs to the bloodstream; therefore, the role of PepT1 is vital to the bioactivity of BPs and needs further investigation [15, 26].

Peptides isolated from a variety of dietary protein sources (e.g., soy bean, common bean, corn, egg white, whey, casein, salmon, and crucian carp) have shown to inhibit intestinal inflammation through multiple molecular mechanisms. These include downregulation of the expression of IL-8, IL-1*β*, IL-6, TNF-*α*, IFN-*γ*, IL-12, and IL-17; upregulation of IL-10; and inhibition of activation of the NF-*κ*B and MAPK pathways via suppression of phosphorylation of p65, ERK1/2, p38, JNK1/2, and Syk signaling molecules [102, 103, 106, 107]. The isolated peptides and their molecular mechanisms of anti-intestinal inflammatory effects are presented in Table 5.

Dietary anti-intestinal inflammatory peptides are short chains of AAs that generally contain 2–10 AAs. The common AAs of these peptides are alanine, valine, leucine, serine, methionine, tyrosine, and phenylalanine [102, 103, 108]. Pepsin and pancreatin are the two most commonly employed proteolytic enzymes to produce anti-inflammatory peptides from various food proteins [103, 109, 110]. The TNF-*α*-treated Caco-2 cell is a widely used human intestinal cell model for the investigation of the anti-inflammatory property of dietary peptides. TNF-*α* activates the both NF-*κ*B and MAPK signaling pathways in Caco-2 cells and thereby produces large quantities of proinflammatory mediators [110]. Excess and uncontrolled production of proinflammatory cytokines plays a vital role in the progression of intestinal inflammation [103].

Numerous recent studies reported that dietary BPs could inhibit the intestinal inflammation through the reduction of proinflammatory mediators. Four peptides, DEDTQAMPFR, MLGATSL, SLSFASR, and MSYSAGF, isolated from egg white exerted anti-inflammatory activities in colitis mouse by inhibiting the production of TNF-*α* and IL-6 as well as reducing the mRNA-expressions TNF-*α*, IL-6, IL-17, IL-1*β*, IFN-*γ*, and MCP-1 [111]. Tripeptide VPY from soybean inhibited IL-8 secretion in Caco-2 cells [108]. The peptide was also found to decrease the mRNA expressions of inflammatory mediators TNF-*α*, IL-6, IL-1*β*, IFN-*γ*, and IL-17 in the peptide-treated mice colon. Dipeptides (CR, FL, HC, lLL, and MK) produced from egg white ovotransferrin, by using pepsin and trypsin hydrolysis, decreased the gene expression of TNF-*α*, IL-8, IL-6, IL-1*β*, and IL-12, while enhancing IL-10 expression, in Caco-2 cells [103]. Crucian carp-derived 178 peptides (<1500 Da) at 50, 100, and 150 *μ*g/mL considerably reduced the secretion of TNF-*α*, IL-6, and IL-1*β* in IEC-6 small intestine cells as well as in dextran sodium sulfate-induced ulcerative colitis mice [106].

Several recent reports demonstrated the role of NF-*κ*B in the pathogenesis of IBD [106]. Activation of NF-*κ*B has been shown to be involved in the IBD patients [112]. The NF-*κ*B and MAPK pathways are two vital proinflammatory signaling pathways that majorly regulate cellular inflammatory responses by secreting various cytokines after activation by various inflammatory stimuli [113]. Transcription factor NF-*κ*B regulates the inflammatory responses by stimulating the production of various proinflammatory cytokines and chemokines. Phosphorylation of I*κ*B by inflammatory stimuli (LPS and TNF-*α*) releases the NF-*κ*B that migrates to the nucleus and activates expression of the numerous genes connected with inflammation [110, 112]. The MAPK family contains three components such as p38 MAPK, ERK1/2, and JNK/SAPK. Phosphorylation of MAPK components stimulates the other kinases and migrates to the nucleus where they induce the transcription of several inflammatory genes and thereby enhance the secretion of proinflammatory mediators [110].

Peptides derived from egg, milk, fish, and beans exhibited the anti-intestinal inflammatory activity through the inhibition of MAPK and NF-*κ*B molecular pathways [102, 103, 110]. A tetrapeptide, IPAV, isolated from whey proteins exhibited anti-inflammatory activity in Caco-2 cells by inhibiting IL-8 expression and by suppression of phosphorylation of p65, ERK1/2, p38, JNK1/2, and Syk signaling molecules [102]. Bean milk- and yogurt-derived LLV, *γ*-E-S-(Me)C, and *γ*-EL inhibited TNF-*α*-induced IL-8 production and gene expression of inflammatory mediators, TNF-*α*, IL-1*β*, IL-8, and IL-6, through the inhibition of phosphorylation of I*κ*B-*α* of NF-*κ*B and JNK of MAPK signaling pathways in Caco-2 cells [113]. Four peptides DEDTQAMPFR, DEDTQAMPF, MLGATSL, and MSYSAGF isolated from egg considerably suppressed the phosphorylation of JNK, p38, and I*κ*B of NF-*κ*B and MAPK signaling pathways and thereby reduced the gene expression of IL-8, IL-1*β*, IL-6, TNF-*α*, and IL-12 in TNF-*α-*stimulated Caco-2 cells [110]. These results indicated that dietary BPs had the potential to treat inflammation or IBD via NF-*κ*B or MAPK or other signaling pathways.

## 7. Conclusions and Further Perspectives

Numerous dietary peptides showed beneficial effects on redox balance and metabolic disorders (obesity, T2D, hypertension, and inflammation). Dietary peptides modulated several molecular mechanisms (e.g., *Keap1*-*Nrf2*-ARE *signaling pathway in oxidative stress*, PPAR-*γ*, C/EBP-*α*, SREBP1 pathway in obesity, IRS-1/PI3K/Akt and AMPK signaling pathways in T2D, ACE inhibition in hypertension, and MAPK in IBD) and thereby exerted positive effects in redox balance and metabolic disorders. Most of the studies are conducted using cell and animal models. Although substantial evidence from cell and animal investigations is available for the BPs as described in this review, scientific evidence from clinical studies is still meager. Hence, more clinical investigations are needed to get in-depth knowledge about the BP's efficacy, absorption, distribution, metabolism, excretion, toxicity, and effect on gut microbiome in the human body in the future. In the future, the benefits and risks of long-term and large-quantity consumption of BPs on human health need to be addressed. The interaction of BPs with other drugs in the human body has to be investigated comprehensively. Additionally, newer technologies are needed to produce BPs cost effectively from dietary sources. The BPs should be produced with consumer-acceptable taste, quality, and stability. Although there are several challenges for future growth, the dietary BPs could be used as health foods in the management/prevention of metabolic disorders (obesity, T2DM, hypertension, and inflammation) and oxidative stress-related diseases (e.g., cancer and IBD). We hope that the BP's industry will have a bright future in the coming years as people are increasingly aware of health benefits of dietary BPs.

## Figures and Tables

**Table 1:** Molecular mechanisms of action of dietary peptides in redox balance.

Dietary protein source	Enzyme used to produce peptides	Peptide sequence or molecular weight	Object	IC_50_/EC_50_ values	Activity/mechanisms of action	Reference
*Ziziphus jujuba* fruits	Papain and trypsin	VGQHTR and GWLK	DPPH, ABTS, and metal chelating assays	—	Peptides scavenged ABTS and DPPH and showed strong metal chelating activity	[114]
Corn gluten	Alcalase	<1 kDa and GLLLPH	H_2_O_2_-induced HepG2	—	Peptides reduced ROS and increased SOD, CAT activities, and GSH levels and GR activity	[115]
Milk protein concentrate	Trypsin	—	Healthy and diabetic rats	—	Peptides enhanced the activities of CAT, SOD and reduced glutathione, glutathione-S-transferase, and GPx	[41]
*Palmaria palmata* macroalgal protein	Corolase PP	SDITRPGGNM	ORAC and FRAP assays	—	Peptide showed strong oxygen radical absorbance capacity and ferric-reducing antioxidant power activity	[116]
Rice bran	Trypsin	YSK	DPPH and reducing power assays	DPPH IC_50_ 0.15 mg/mL	Peptide exhibited high DPPH free radical scavenging activity and reducing power	[1]
Nile tilapia skin gelatin	Ginger protease	GPA	H_2_O_2_-induced IPEC-J2 cell	—	GPA activated the expression of antioxidant response element-driven antioxidant enzyme genes HO-1, NAD(P)H quinone oxidoreductase-1, and glutamyl cysteine ligase modulator and suppressed ROS production	[37]
Manchurian walnut (*Juglans mandshurica* Maxim.)	Alkaline protease	<3 kDa	Mice	—	Peptides increased the antioxidant capacity by enhancing SOD, GSH-Px, and CAT activities and reducing the MDA content	[43]
Soybean	Alcalase	<3 kDa	H_2_O_2_-incuded oxidative stress in Caco-2 cell and DPPH assay	DPPH IC_50_ 2.56 mg/mL	Peptides displayed DPPH radical scavenging activity and decreased intracellular ROS and stimulated the antioxidant enzymes CAT, GP, and GR	[42]
Oyster (*Crassostrea rivularis*) meat	Alcalase	<3 kDa	Normal male mice	—	Peptides showed antioxidant capacity by increasing the activities of GSH-Px, SOD, and CAT and reducing MDA levels	[44]
Buffalo casein	—	YFYPQL	H_2_O_2_ induced Caco-2 cell and ABTS and ORAC assays	—	YFYPQL showed antioxidant and inhibited ROS generation and decreased cellular oxidative products, MDA, and protein carbonyls and increased CAT, SOD, and GPx by stimulating Nrf2 stress signaling and scavenged ABTS and ORAC free radicals	[40]
Wheat germ protein	Alcalase, pepsin, and proteinase K	TVGGAPAGRIVME, VGGIDEVIAK, GNPIPREPGQVPAY, SGGSYAD ELVSTAK, and MDATALHYENQK	ABTS assay	—	Peptides exhibited strong ABTS radical scavenging activity	[117]
Carp (*Cyprinus carpio*) skin gelatin	Protamex	—	Healthy adult Wistar rats	—	Peptides showed antioxidant activity by increasing the glutathione reductase activity	[118]
Sesame (-icum L.) seed protein	Alcalase and trypsin	RDRHQKIG, TDRHQKLR, MNDRVNQGE, RENIDKPSRA, SYPTECRMR, GGVPRSGEQEQQ, and AGEQGFEYVTFR	DPPH and ABTS assays	DPPH IC_50_ 0.105 and ABTS IC_50_ 0.004 mg/mL	SYPTECRMR exhibited the highest DPPH and ABTS free radical scavenging antioxidant activity	[34]
Finger millet	Trypsin	STTVGLGISMRSASVR and TSSSLNMAVRGGLTR	DPPH assay	DPPH 75–80%	Peptides exhibited DPPH and ABTS radical scavenging activities by interaction of serine and threonine residues of peptides with free radicals	[119]
Potato	—	Dipeptide IF	SHR rats	—	Peptides increased the antioxidant enzymes HO-1, GPx, SOD, and peroxiredoxin 2 through the Akt pathway to regulate Nrf2 activity and prevented Nrf2 degradation by Akt activation and GSK-3*β* phosphorylation	[33]
*Mytilus Coruscus* mussel	Trypsin	<1 kDa	H_2_O_2_-induced HUVEC and OH, O_2_, and ferric-reducing assays	—	Peptides reduced the accumulation of ROS and MDA production and increased the levels of the SOD, CAT, and GSH-Px cellular antioxidant capacities through regulating the Nrf2-driven antioxidant defense mechanisms. Peptides showed strong OH, O_2_ radical scavenging activities and ferric-reducing power	[46]
Mackerel (*Scomber japonicus*) muscle	Protamex	ALSTWTLQLGSTSFSASPM	DPPH assay	DPPH 36.34%	Peptide showed strong DPPH radical scavenging activity with 36% inhibition	[120]
Soft-shelled turtle	Neutrase, papain, proteinase, pepsin, and trypsin	EDYGA	HepG2 cells	—	EDYGA modulated the Nrf2/ARE pathway by enhancing the Nrf2 level via Nrf2 stabilization and decreasing the level of Keap1 and glutamate residues of EDYGA bound to the Arg 415 of Kelch domain receptor pocket	[45]
Foxtail millet (*Setaria italica*) prolamins	Alcalase	PFLF and IALLIPF	H_2_O_2_-induced human keratinocyte HaCaT cells	—	Peptides decreased the production of ROS and MDA and enhanced the GSH level	[121]
Krill	Pepsin	AMVDAIAR	H_2_O_2_-stimulated hepatocytes	DPPH IC_50_ 0.87 mM	Peptide reduced oxidative stress by enhancing SOD, CAT, and GPx. Peptide increased Nrf2 and HO-1 expression and activated Nrf2/HO-1 by activating the ERK pathway	[38]
Watermelon seed protein	Alcalase	RDPEER	H_2_O_2_-induced oxidative stress in HepG2 cells	—	RDPEER reduced the oxidative stress by increasing CAT, SOD, and GSH-Px, and reducing MDA production and ROS accumulation	[39]
Scallop (*Patinopecten yessoensis*) shellfish	Pepsin, dispase, and alcalase	<3 kDa	DPPH, HO^∗^, and ABTS assays and H_2_O_2_-induced PC-12 cells	DPPH EC_50_ 1.30–2.40, ABTS EC_50_ 0.75–1.98, and OH EC_50_ 1.07–1.43 mg/mL	Peptides scavenged the free radicals of DPPH, HO^∗^, ABTS, and inhibited ROS accumulation	[122]
Milk casein	—	ARHPHPHLSFM, AVPYPQR, NPYVPR, and KVLPVPEK	Peroxide-induced oxidative stress Caco-2 cells	—	Peptides enhanced the expression of SOD1, Trx1, TrxR1, GR, and NQO1 by activating the Keap1-Nrf2 pathway. Peptides inhibited the interaction between Keap1 and Nrf2, by binding to Nrf2 in the Keap1 pocket and increased antioxidant enzyme expression	[32]
Corn gluten meal	Fermentation mice with *Bacillus subtilis* MTCC5480 (BS5480)	<10 kDa	Aging rats	—	Peptides increased activities of total SOD, CAT, GPx, and total antioxidant capacity and decreased MDA	[31]
*Moringa oleifera* seeds	Flavor protease	GY, PFE, YTR, FG, QY, IN, SF, SP,YFE, IY, and LY	H_2_O_2_ induced oxidative damage in Chang liver cells and DPPH and ABTS assays	DPPH EC_50_ 0.75–2.28 mg/mL and ABTS EC_50_ 0.32–1.03 mg/mL	Peptides exhibited strong scavenging activities on free radicals DPPH and ABTS^+^. SF and QY scavenged ROS by increasing SOD and CAT and reducing MDA	[25]
Ginger	Pepsin and trypsin	VTYM	DPPH and ABTS assays	EC_50_ of DPPH 19.9 ± 2.1 and ABTS 24.0 ± 3.7 *μ*mol/L	VTYM showed potent DPPH and ABTS radical scavenging activity	[9]
Snakehead (*Channa argus*) soup	Pepsin and pancreatin	IVLPDEGK, PGMLGGSPPGLLGGSPP, SDGSNIHFPN, and SVSIRADGGEGEVTVFT	DPPH and Fe^2+^ chelating assays and H_2_O_2_ induced HepG2 cells	DPPH IC_50_ 1.39 mM and Fe^2+^ chelating ability IC_50_ 4.60 mM	Peptides exhibited strong DPPH and Fe^2+^ chelating ability and molecular docking indicated that peptides can bind to the active site of Keap1 and thereby activate the cellular antioxidation Keap1-Nrf2 pathway	[3]
Silver carp muscle	Papain and alcalase	<1 kDa and LVPVAVF	H_2_O_2_ induced oxidative stress Caco-2 cells and DPPH assay	DPPH EC_50_ 0.65 mg/mL	Peptides showed antioxidant activity by enhancing the activity of SOD, CAT, and GSH-Px and reduced ROS and showed strong DPPH scavenging activity	[12]

ARE: antioxidant response element; ATBS: 2,2′-azino-bis (3-ethylbenzothiazoline-6 sulphonic acid) diammonium salt; Akt: protein kinase B; CAT: catalase; DPPH: 2,2-diphenyl-1-picrylhydrazyl; ERK: extracellular signal-regulated kinases; FRAP: ferric reducing antioxidant power; GPx: glutathione peroxidase; GSH: glutathione; GR: glutathione reductase; H_2_O_2_: hydrogen peroxide; HO-1: heme oxygenase 1; IC_50_: 50% inhibitory concentration; ROS: reactive oxygen species; SHR: spontaneously hypertensive rats; SOD: superoxide dismutase; MDA: malondialdehyde; NQO1: NAD(P)H quinine dehydrogenase 1; Nrf2: nuclear factor erythroid 2-related factor; HUVEC: human umbilical vein endothelial cells; Keap 1: Kelch-like ECH-associated protein 1; HO: heme oxygenase; Trx1: thioredoxin 1; TrxR1: thioredoxin reductase 1; ORAC: oxygen radical absorbance capacity.

**Table 2:** Mechanisms of action of antiobesity peptides derived from various food sources.

Dietary protein source	Enzyme used to produce peptides	Peptide sequence or molecular weight	Object	Dose & duration	Activity/mechanisms of action	Reference
Soy protein	Flavourzyme	<1300 Da	3T3-L1 preadipocytes	100 ppm for 8 d	Peptides reduced GPDH activity and inhibited adipogenesis by affecting the expression of PPAR-*γ* and CCAAT/enhancer binding protein-*α*	[123]
Smooth hound (*Mustelus mustelus*) muscle protein	Alkaline crude enzymes from *M. mustelus* intestines	200–2500 Da	Rats	0.5 mL (10 mg/mL)/day/kg BW for 21 d	Peptides reduced BW and food intake	[5]
Soy protein	Flavourzyme	ILL, LLL, and VHVV	3T3-L1 adipocytes	4 ppm for 72 h	Peptides exhibited lipolysis-stimulating activity	[52]
Canola protein	Alcalase, chymotrypsin, pepsin trypsin, and pancreatin	<1–10 kDa	C3H10T1/2 murine mesenchymal stem cells	60–100 *μ*g/mL for 24 h	Peptides showed antiobesity effects by inhibiting PPAR*γ* expression and pancreatic lipase	[54]
Common bean	Alcalase, bromelain, and pepsin-pancreatin	<1 kDa	Mature adipocytes 3T3-L1	0.1, 1, 10, and 100 *μ*g/mL for 48 h	Peptides inhibited lipid accumulation (28%)	[53]
Salmon protein	—	—	Placebo-controlled, randomized clinical study	16 g for 42 days	Peptide supplementation for 42 days reduced the body mass index by 5.6% in overweight subjects	[59]
Ark shell (*Scapharca subcrenata*) protein	Pepsin	<1 kDa	Mouse mesenchymal stem cells	400 *μ*g/mL for 7 d	Peptides inhibited intracellular lipid accumulation and enhanced lipolysis. Peptides inhibited adipogenesis by downregulating the adipocyte-specific protein expression including PPAR-*γ*, C/EBP-*α*, SREBP-1c, downstream lipoprotein lipase, and FAS expression	[51]
Yellow catfish protein	Alcalase	—	HFD fed mice	500, 250 and 125 mg/kg BW for 84 d	Peptides exhibited anti-obesity effects	[124]
Sardinella (*Sardinella aurita*) protein	*Bacillus subtilis* A26 and *Bacillus amyloliquefaciens* An6	150–900 Da	Wistar rats fed high caloric diet	400 mg/kg BW for 10 weeks	Peptides reduced BW gain, food intake, and the relative epididymal adipose tissue and decreased the pancreatic lipase activity	[2]
Alaska pollack protein	Pepsin and pancreatin	—	Rats	0, 100, and 300 mg/kg BW for 3 d	Peptides reduced white adipose tissue weight and food intake	[56]
Tuna skin	Subcritical water hydrolysis	—	3T3-L1 preadipocytes and obese mice fed HFD	300 mg/kg/day for 8 weeks	Peptides decreased HFD-induced BW gain and inhibited the expression of C/EBP-*α*, PPAR-*γ* and adipocyte protein 2	[55]
Camel whey protein	Pepsin, trypsin, and chymotrypsin	<10 kDa	In vitro assays	50 *μ*L for 30 min	Peptides exhibited antiobesity effects by inhibiting pancreatic lipase and cholesteryl esterase enzymes	[125]
Skate (*Raja kenojei*) skin collagen	—	1050 Da	HFD-fed mice	100, 200, or 300 mg/kg BW for 8 weeks	Peptides showed antiobesity effects by reducing BW gain and visceral adipose tissue and improved the dyslipidemia via regulating hepatic lipid metabolism and AMPK	[126]
Camel milk	Alcalase, bromelin, and papain	<10 kDa	In vitro	—	Peptides inhibited the porcine pancreatic lipase	[24]
Kefir	—	>30 kDa, 3–30 kDa, and <3 kDa	HFD-induced obese rats	164 mg/kg BW daily for 8 weeks	Peptides blocked lipogenesis by reducing FAS and increased p-acetyl-CoA carboxylase. Peptides enhanced FA oxidation via increasing the expressions of phosphorylated AMPK, PPAR-*α*, and hepatic carnitine palmitoyltransferase-1	[127]
*Spirulina platensis* protein	Trypsin, alcalase, pepsin, papain, and protamex	NALKCCHSCPA, LNNPSVCDCDCMMKAAR, NPVWKRK, and CANPHELPNK	3T3-L1 preadipocytes	1 mg/mL for 48 h	Peptides exhibited antiobesity effects by inhibiting lipase (72%) and 3T3-L1 preadipocytes (72.7–88.1%) and decreased triglyceride accumulation	[8]
Quinoa protein	Papain, pepsin, and pancreatin	FGVSEDIAEKLQAKQDERGNIVL, AEGGLTEVWDTQDQQF, YIEQGNGISGLMIPG, AVVKQAGEEGFEW, and HGSDGNVF	3T3-L1 cells	0–1600 *μ*g/mL for 48 h	Peptides inhibited lipid accumulation during differentiation and suppressed cell differentiation through PPAR-*γ*	[128]
*Spirulina platensis* protein	Pepsin	<10 kDa	HFD-fed mice	2 g/kg BW/d for 4 weeks	Peptides showed antiobesity effects reducing BW, lowering serum glucose, and total cholesterol through modulation of expressions of Acadm, Retn, Fabp4, Ppard, and Slc27a1 in the brain and liver	[57]
Pea (*Pisum sativum L*.) seed proteins	Pepsin and pancreatin	<6 kDa	3T3-L1 murine preadipocytes	0, 1, 2, 4, and 6 mg/mL for 24 h	Peptides stimulated adipocyte differentiation through upregulation of PPAR-*γ* expression and ligand activity	[48]
Walleye pollock skin collagen	Flavourzyme and alcalase	500–5000 Da	HFD-fed C57BL/6J mice	800 mg/kg BW for 8 weeks	Peptides inhibited weight gain, adipocyte growth, adipose tissue accumulation, and liver weight and reduced the blood-lipid level	[58]
Blue mussel	Pepsin	<1 kDa	Mouse mesenchymal stem cells	100, 200, and 400 *μ*g/mL for 7 or 21 d	Peptides enhanced lipolysis and downregulated adipogenic transcription factors including PPAR*γ*, CCAAT/enhancer-binding protein-*α*, and SREBP-1	[10]
Hazelnut (*Corylus heterophylla Fisch*) protein	Alcalase	Arg-Leu-Leu-Pro-His	3T3-L1 adipocytes	0, 20, 40, and 80 mM for 8 d	Peptides decreased adipogenesis by downregulating the expression of PPAR-*γ*, C/EBP-*α*, aP2, SREBP-1c, FAS, ACC1, and 3-hydroxy-3-methylglutaryl-CoA reductase	[13]
Milk *β*-casein	Trypsin	7 kDa	HepG2 cells and humans	5 mg/mL for 24 h	Casein oligopeptide increased FGF-21	[129]

ACC1: acetyl-CoA carboxylase 1; AMPK: AMP-activated protein kinase; aP2: adipocyte fatty acid-binding protein 2; BW: body weight; C/EBP-*α*: CCAAT/enhancer binding protein alpha; FAS: fatty acid synthase; FGFs: fibroblast growth factors; HFD: high-fat diet; PPAR-*γ*: peroxisome proliferator-activated receptor-*γ*; SREBP-1: sterol regulatory element-binding protein 1.

**Table 3:** Molecular mechanisms of action of antidiabetic peptides isolated from various dietary sources.

Dietary protein source	Enzyme used to produce peptides	Peptide sequence or molecular weight	Object	IC_50_/EC_50_ values	Activity/mechanisms of action	Reference
Rice bran	Umamizyme G and bioprase SP	Dipeptides LP and IP	DPP-IV inhibition assay	DPP-IV IC_50_2.3 ± 0.1 mg/mL	Peptides sowed strong DPP-IV inhibition activity	[130]
Egg white albumin	Alcalase	KLPGF	*α*-Glucosidase and *α*-amylase inhibitory assays	*α*-Glucosidase inhibitory IC_50_59.5 ± 5.7 *μ*M and *α*-amylase inhibitory IC_50_ 120 *μ*M	KLPGF exhibited strong antidiabetic potential by inhibiting *α*-glucosidase and *α*-amylase activities	[66]
Casein	Prolyl oligopeptidase	FLQP	DPP-IV inhibition assay	DPP-IV IC_50_65.3 ± 3.5 *μ*M	FLQP exhibited DPP-IV inhibition activity	[70]
Bovine and porcine meat proteins	Papain and pepsin	PPL	DPP-IV inhibition assay	DPP-IV IC_50_ 390.14 *μ*M	Peptides showed DPP-IV inhibition	[131]
Porcine skin	Alcalase and flavourzyme	—	Streptozotocin-induced diabetic rats	—	Peptides improved glucose tolerance and inhibited DPP-IV activity and enhanced GLP-1 and the insulin level	[132]
Egg yolk	Proteinase from Asian pumpkin	LAPSLPGKPKPD	DPP-IV and *α*-glucosidase assays	DPP-IV IC_50_ 361.5 *μ*mol/L and *α*-glucosidase IC_50_ 1065.6 *μ*mol/L	Peptides showed DPP-IV and *α*-glucosidase inhibitory activities	[67]
Halibut and tilapia skin gelatin	Flavourzyme	SPGSSGPQGFTG,GPVGPAGNPGANGLN, PPGPTGPRGQPGNIGF, IPGDPGPPGPPGP, LPGERGRPGAPGP, and GPKGDRGLPGPPGRDGM	Streptozotocin-induced diabetic rats	—	Peptides improved glucose tolerance through DPP-IV inhibition and GLP-1 secretion enhancement	[73]
*Styela clava*	Protamex	—	Patients with diabetes	—	Peptides exhibited a decreased hemoglobin A1c and plasma insulin levels	[78]
Black bean	Alcalase	AKSPLF, LSKSVL, FEELN, and PHL	Caco-2 cell and rats	—	Peptides showed antidiabetic effects by blocking GLUT2 and SGLT1 and reduced glucose absorption and postprandial glucose and blood glucose	[133]
Wheat	Bacterial protease	770–77740 Da	GLUTag cells and rats	—	Peptides improved hyperglycemia via activating GLP-1 secretion via stimulation of the calmodulin-dependent kinase II pathway mediated by G protein-coupled receptor family C group 6 subtype A	[134]
Atlantic cod (*Gadus morhua*) meat	Protamex	<2000 Da	41 healthy individuals	—	Peptides decreased the postprandial insulin	[77]
Oat globulin	Trypsin	OGb, LQAFEPLR, and EFLLAGNNK	Caco-2 cell	DPP-IV IC_50_ OGb 188.1 *μ*g/mL and LQAFEPLR IC_50_ 141.7 *μ*M	Peptides showed potent inhibition on DPP4 and *α*-glucosidase activity and reduced DPP4 protein expression and upregulated the expressions of *α*-glucosidase, GLUT2, and GLUT5	[135]
Milk whey protein	Protease	<5000 Da	21 prediabetic humans	—	Peptides (1400 or 2800 mg/kg BW) decreased under glucose curve and showed a minor insulinotropic and reduced HbA1c	[76]
Egg white	Thermolysin and pepsin	IRW	TNF-*α*-treated L6 rat skeletal muscle cells	—	IRW reduced glucose uptake and enhanced insulin receptor activation and improved insulin sensitivity by inhibiting p38 and JNK1/2 activation	[23]
Boarfish (*Capros aper*) protein	Alcalase and flavourzyme	<2 kDa	BRIN-BD11 and GLUTag cells and mice	DPP-IV inhibitory activity IC_50_ 1.18 mg/mL	Peptides increased insulin secretion and inhibited DPP-IV activity. Peptides increased insulin levels and reduced glucose concentration	[72]
Blue whiting (*Micromesistius poutassou*) muscle protein	Alcalase and flavourzyme	<5 kDa	GLUTag cells, BRIN-BD11 cells, 3T3-L1 adipocytes, DPP-IV assay, and mice	DPP-IV inhibitory activity IC_50_1.28 ± 0.04 mg/mL	Peptides showed being antidiabetic via DPP-IV inhibitory activity, increasing insulin-stimulated glucose, stimulating insulin secretion and GLP-1, and decreasing glucose	[71]
Potato protein	Alcalase	DIKTNKPVIF	Diabetic mice	—	Peptides showed antidiabetic effects via regulation of blood glucose, plasma total glycerol, total cholesterol, insulin, and HbA1c	[6]
Spirulina platensis	—	GVPMPNK, RNPFVFAPTLLTVAAR, and LRSELAAWSR	*α*-Amylase, *α*-glucosidase, and DPP-IV assay	*α*-Amylase IC_50_ 313.6 *μ*g/mL, *α*-glucosidase IC_50_ 134.2 *μ*g/mL, and DPP-IV IC_50_ 167.3 *μ*g/mL	LRSELAAWSR exhibited strong inhibitory activity on *α*-amylase, *α*-glucosidase, and DPP-IV	[68]
Beans (*Phaseolus vulgaris* L.)	Pepsin and pancreatin	<3 kDa	Wistar rats and mice and in vitro assays	*α*-Amylase 16.9–89.1% and *α*-glucosidase inhibition 34.4–89.2%	Fractions inhibited *α*-amylase and *α*-glucosidase. Fractions showed both hypoglycemic and antihyperglycemic activities	[136]
Soy protein	Papain, trypsin, and alkaline proteinase	LLPLPVL, SWLRL, and WLRL	*α*-Glucosidase inhibitory assay	*α*-Glucosidase IC_50_ 162.2–237.4 *μ*mol/L	Peptides showed strong *α*-glucosidase inhibitory activity	[63]
Sea cucumber (*Holothuria nobilis*)	Mixture of papain and protamex	203–1907 Da	Type II diabetic rats induced by streptozotocin	—	Peptides (200 and 400 mg/kg BW) decreased fasting blood glucose. Peptides showed antidiabetic effects by increasing the expressions of PI3K, p-Akt, p-GSK-3*β*, and GLUT2/4 signaling pathways and decreasing the expression of p-IRS1	[73]
Largemouth bass (*Micropterus salmoides*)	Pepsin, trypsin, and chymotrypsin	ICY	DPP-IV inhibitory assay	DPP-IV IC_50_ 0.73 mM	ICY had strong DPP4 inhibitory activities	[137]
Zebra blenny (*Salaria basilisca*) protein	Crude alkaline protease extract from zebra blenny	>30 kDa	DPP-IV inhibitory assay	DPP-IV IC_50_ 71 *μ*g/mL	Fraction showed *α*-amylase inhibitory activity	[64]
Walnut (*Juglans mandshurica* Maxim)	Alcalase	LPLLR	Hepatic HepG2 cells and in vitro assays	Inhibiting *α*-glucosidase 50.12% and *α*-amylase 39.08% at 2000 *μ*M	LPLLR inhibited *α*-glucosidase and *α*-amylase and improved hepatic insulin resistance via enhancing glycogen synthesis and glucose uptake and reduced gluconeogenesis via activating the IRS-1/PI3K/Akt and AMPK pathways	[75]
Quinoa protein	Bromelain, chymotrypsin, and Pronase E	QHPHGLGALCAAPPST	*α*-Glucosidase and DPP-IV inhibitory assays	DPP-IV IC_50_ 0.72–1.12 mg/mL and *α*-glucosidase IC_50_ 1.0–1.45 mg/mL	Peptides showed antidiabetic effects by inhibiting DPP-IV and *α*-glucosidase	[62]
Corn germ protein	Alcalase, trypsin, and flavourzyme	<2–10 kDa	In vitro assays	Inhibiting *α*-amylase 71.3%, *α*-glucosidase 37.1%, and DPP-IV 45.9%	Peptides showed strong *α*-amylase, *α*-glucosidase, and DPP-IV inhibition	[61]
Sea cucumber (*Stichopus japonicus*)	Pepsin, trypsin, and chymotrypsin	<3 kDa	3T3-L1 and Hep G2 cells	DPP-IV IC_50_ 0.51–0.52 mg/mL	Peptides improved glucose uptake and DPP-IV inhibitory activity	[7]
*α*-Lactalbumin-rich whey proteins	Trypsin	LDQWLCEKL	DPP-IV inhibitory activity	DPP-IV inhibition IC_50_ 131 *μ*M	LDQWLCEKL exhibited DPP-IV inhibition with a noncompetition	[138]
*Palmaria palmata*	Alcalase and flavourzyme	<1–5 kDa	Streptozotocin-induced diabetic mice	—	Peptides showed antidiabetic effects by reducing blood glucose and increasing insulin and improved terminal oral glucose tolerance and fasting blood glucose	[139]
Atlantic salmon (*Salmo salar*) skin	Trypsin	LDKVFR	DPP-IV inhibitory activity assay	DPP-IV inhibition IC_50_ 128.7 *μ*M	LDKVFR showed DPP-IV inhibition	[14]
Millet proteins	Papain	NDWHTGPLS and TYPHQQPPILT	DPP-IV inhibition assay	DPP-IV inhibition 75.72%	Peptides inhibited DPP-IV and occupied DPP-IV active center (S1 and S2 subsites) via H-bond and *π* − *π*	[21]

Akt: protein kinase B; AMPK: AMP-activated protein kinase; DPP-IV: dipeptidyl peptidase-IV; GLP-1: glucagon-like peptide-1; GLUT: glucose transporter; HbA1c: glycosylated hemoglobin; IC_50_: 50% inhibitory concentration; STZ: streptozotocin; PI3K: phosphatidylinositol 3-kinase; p-Akt: phosphorylated protein kinase B; p-IRS1: phosphorylated insulin receptor substrate-1; IRS-1: insulin receptor substrate-1; JNK: c-Jun N-terminal kinase.

**Table 4:** Molecular mechanisms of action of antihypertensive peptides isolated from various food sources.

Dietary protein source	Enzyme used to produce peptides	Peptide sequence or molecular weight	Object	IC_50_/EC_50_ values	Activity/mechanisms of action	Reference
Pea protein	Thermolysin	<3 kDa	SHR and clinical trial	—	Peptides (100 and 200 mg/kg BW) reduced SBP	[140]
Casein	—	VPP and IPP	Clinical trial	—	VPP and IPP improved central blood pressure and arterial stiffness	[81]
Pistachio kernel	Pepsin and trypsin	ACKEP	ACE inhibition assay	ACE IC_50_ 126 *μ*M	ACKEP inhibited ACE by binding with ACE active site	[88]
Chum salmon (*Oncorhynchus keta*) skin	Trypsin	GLPLNLP	ACE inhibition assay and SHRs	ACE IC_50_ 18.7 *μ*M	GLP exhibited ACE inhibition and antihypertensive effect by decreasing SBP	[94]
Skate (*Okamejei kenojei*) skin	Alcalase and protease	LGPLGHQ and MVGSAPGVL	ACE inhibition assay and SHRs	ACE IC_50_ 3.09–4.22 *μ*M	Peptides inhibited ACE and decreased SBP and inhibited vasoconstriction via PPAR-*γ* expression, activation, and phosphorylation of eNOS in lungs	[95]
Egg white ovotransferrin	Thermolysin and pepsin	IQW and LKP	SHRs	—	Peptides reduced mean blood pressure	[22]
Cuttlefish (*Sepia officinalis*) muscle	Crude enzymes from *B. mojavensis* and cuttle fish hepatopancreas	VELYP, AFVGYVLP, and EKSYELP	ACE inhibition assay and SHRs	ACE IC_50_ 5.22 *μ*M	VELYP showed strong ACE inhibition through a noncompetitive inhibition and had antihypertensive effects by decreasing SBP	[96]
Potato and rapeseed	Alcalase and potato autolysis	SLVSPSAAAAAAPGGS and KKRSKKKSFG	Goldblatt rat with hypertension and ACE inhibition	ACE IC_50_ 324 *μ*g/mL and 156 *μ*g/mL	Peptides inhibited ACE and exhibited antihypertensive effects by reducing SBP	[85]
Rice bran protein	Trypsin	YSK	ACE inhibition assay	ACE IC_50_ 76 mM	YSK showed ACE inhibition through the formation of hydrogen bonds with active pockets of human ACE	[1]
Sipuncula (*Phascolosoma esculenta*)	Pepsin and trypsin	RYDF, YASGR and GNGSGYVSR	ACE inhibition assay and SHRs	ACE IC_50_ 235 *μ*M, 185 *μ*M, and 29 *μ*M	Three peptides inhibited ACE noncompetitively. GNGSGYVSR (5 mg/kg BW) showed antihypertensive effect by decreasing SBP	[97]
Lentil seeds (*Lens culinaris* var.)	Savinase	LLSGTQNQPSFLSGF, NSLTLPILRYL, and TLEPNSVFLPVLLH	ACE inhibition assay	ACE IC_50_ 44–120 *μ*M	Inhibited ACE through interaction by hydrogen bonds with three ACE residues of the catalytic site	[91]
Bighead carp muscle	Pepsin	YNLKERYAAW and YNRLPEL	ACE inhibition assay	ACE IC_50_ 1.35–3.42 *μ*M	Peptides inhibited ACE activity	[98]
Bovine casein	Pepsin and trypsin	YQKFPQYLQY	ACE inhibition assay and SHRs	ACE IC_50_ 11.1 *μ*M	Peptide inhibited ACE via competitive inhibition and exhibited antihypertension by decreasing SBP	[82]
Hazelnut (*Corylus heterophylla* Fisch.)	Alcalase	AVKVL, YLVR, and TLVGR	ACE inhibition assay and SHRs	ACE IC_50_ 15.42–249.3 *μ*M	Peptides inhibited ACE activity via a noncompetitive mode via the formation of cation–pi interactions and YLVR reduced SBP	[89]
Egg white from ostrich	Alkaline hydrolysis	YV	ACE inhibition assay	ACE IC_50_ 63.97 *μ*g/mL	YV showed ACE inhibition by binding to S1 and S2 ACE pocket sites via hydrogen bonds	[93]
Soybean	*Pediococcus pentosaceus* SDL1409	EDEVSFSP, SRPFNL, RSPFNL, and ENPFNL	ACE inhibition assay	ACE IC_50_ 0.131–0.811 mg/mL	Peptides inhibited ACE via essential N-terminal sequence and amino acid position	[84]
Shrimp (*Pandalus borealis*) protein	—	—	Randomized, double-blind, placebo-controlled, 8-week clinical study	—	Peptides (1200 mg/d) reduced the blood pressure due to a reduction of angiotensin II levels	[79]
Beef (Bos taurus coreanae) myofibrillar proteins	Alkaline-AK and papain	LIVGIIRCV	ACE inhibition assay and SHRs	—	Peptides (400 and 800 mg/kg BW) inhibited ACE by 74.29% and decreased SBP	[99]
Milk	Fermented using *L. delbrueckii* QS306	LPYPY	ACE inhibition assay	ACE IC_50_ 12.87 *μ*g/mL	LPYPY inhibited ACE with IC_50_ 12.87 *μ*g/mL	[80]
Mung bean protein	Bromelain	LPRL, YADLVE, LRLESF, HLNVVHEN, and PGSGCAGTDL	ACE inhibition assay and SHRs	ACE IC_50_ 5.39–1912 *μ*M	Peptides showed ACE inhibition and reduced SBP	[90]
Seahorse (Hippocampus abdominalis)	Protamex	APTL, CNVPLSP, and PWTPL	ACE inhibition assay and SHRs	ACE IC_50_ 0.044 *μ*M	Peptides exhibited antihypertension by lowering blood pressure via vasodilation and ACE inhibition	[92]
Black cumin seed	*α*-Chymotrypsin	VTPVGVPK	ACE inhibition assay	ACE IC_50_ value 1.8 *μ*M	VTPVGVPK inhibited ACE via a noncompetitive inhibition	[86]
Quinoa protein	Chymotrypsin	QHPHGLGALCAAPPST	ACE inhibition assay	—	Peptide displayed ACE inhibition by binding to ACE active hotspots	[62]
White turmeric, turmeric, and ginger proteins	Pepsin and trypsin	VTYM, RGPFH, AEPPR, GSGLVP, KM, SPV, CACGGV, DVDP, CGVGAA, HVVV, and RSC	ACE inhibition assay	ACE IC_50_ 16.4–36.5 *μ*M	Peptides showed ACE inhibition	[9]
*Coix* prolamin	Pepsin	VDMF	ACE inhibition assay	ACE IC_50_ 382.28 *μ*M	VDMF reduced ACE and AT1R expression in AngII-injury HUVECs	[87]
Wheat gluten	Alcalase and PaproA	SAGGYIW and APATPSFW	ACE inhibition assay	ACE IC_50_ 0.002–0.036 mg/mL	Peptides and negatively charged amino acids inhibited ACE via modulating ionic and hydrophobic interactions on ACE catalytic sites	[83]

ACE: angiotensin-converting enzyme; BW: body weight; IC_50_: 50% inhibitory concentration; SBP: systolic blood pressure; SHR: spontaneously hypertensive rat; PPAR-*γ*: peroxisome proliferator-activated receptor *γ*.

**Table 5:** Mechanisms of action of anti-inflammatory peptides isolated from various food sources.

Dietary protein source	Enzyme used to produce peptides	Peptide sequence or molecular weight	Object	Dose & duration	Activity/mechanisms of action	Reference
Soy bean	—	150–500 Da	Pigs with DSS-induced colitis	250 mg/kg BW for 5 d	Peptides decreased TNF and IL-6 levels and inhibited IFN-*γ*, IL-1*β*, and TNF expression	[141]
Soy bean	—	VPY	Caco-2 cells and mouse of DSS-induced colitis mice	0.1, 1, 2, and 4 mM for 2 h and 10 and 100 mg/kg BW for 14 d	VPY inhibited IL-8 secretion and reduced the expressions of TNF-*α*, IL-6, IL-1*β*, IFN-*γ*, and IL-17	[108]
Salmon	—	<1000 Da	DSS-induced colitis in rats	3.5% in diet for 29 d	Peptides reduced inflammation by reducing IL-6 and IL-1*β* expressions	[142]
Egg shell membrane	Alcalase and protease S	—	TNF-*α* induced Caco-2 cells and DSS-treated colitis mice	0.001, 0.01, 0.5, and 0.1 mg/mL for 2 h	Peptides inhibited IL-8 secretion and decreased TNF-*α* and IL-6 levels	[143]
Milk casein	Bacterial food-grade enzyme	<5000 Da	TNF-*α*-induced Caco-2 cells and ex vivo porcine colonic tissue system	0.01, 0.02, 0.05, 0.1, 0.5, 1, 2.5, and 5 mg/mL for 24 h	Peptides reduced IL-8 by 66–68% and reduced IL-1*α*/*β*, IL-8, TGF-*β*, and IL-10 expression	[107]
Rat collagen	Pepsin	—	DSS-induced colitis in mice	100 mg/kg BW from days 6 to 15	Peptides reduced IL-1*β* and IL-6 expression	[144]
Egg white	Pepsin and pancreatin	—	TNF-*α*-induced inflammation in Caco-2 cells and DSS-induced colitis mice	0.05, 0.1, 0.5, 1, and 2.5 mg/mL for 2 h	Peptides inhibited IL-8 secretion and decreased expression of TNF-*α*, IL-6, IL-1*β*, IFN-*γ*, and IL-17 and enhanced IL-10 expression and decreased the expression of TNF-*α*, IL-6, IL-1*β*, IFN-*γ*, and IL-17	[145]
Egg white ovotransferrin	Pepsin and trypsin	CR, FL, HC, LL, and MK	TNF-*α*-induced Caco-2 cells	0.05, 0.1, 0.5, 1, 2.5, and 5 mg/mL for 2 h	Peptides decreased expression of TNF-*α*, IL-8, IL-6, IL-1*β*, and IL-12, and enhanced IL-10 expression. CR and HC attenuated intestinal inflammation by inhibiting NF-*κ*B and MAPK pathways	[103]
Milk whey	Pronase	IPAV	TNF-*α* induced Caco-2 cells	2.5 and 5 mg/mL for 1 h	IPAV showed anti-inflammatory effect by inhibiting IL-8 expression and suppressing phosphorylation of p65, ERK1/2, p38, JNK1/2, and Syk signaling	[102]
Egg white	—	DEDTQAMPFR, MLGATSL, SLSFASR, and MSYSAGF	DSS-induced colitis mice	50 or 150 mg/kg/day for 14 d	Peptides inhibited the local production of TNF-*α* and IL-6 and reduced the expression of TNF-*α*, IL-6, IL-17, IL-1*β*, IFN-*γ*, and MCP-1	[111]
Common bean (*Phaseolus vulga* L.) milk and yogurts	Pepsin and pancreatin	*γ*-E-S-(Me)C, *γ*-EL, and LLV	TNF-*α*-induced Caco-2 cells	0.5 mg/mL for 2 h	Peptides inhibited TNF-*α*-induced IL-8 production via inhibiting activation of NF-*κ*B and MAPK pathways	[113]
Egg white	Pancreatin	DEDTQAMPFR, DEDTQAMPF, MLGATSL, and MSYSAGF	TNF-*α*-induced Caco-2 cells	0.1, 0.25, or 0.5 mg/mL for 2 h	Peptides downregulated the expression of IL-8, IL-1*β*, IL-6, TNF-*α*, and IL-12 and upregulated IL-10 expression via inhibiting NF-*κ*B and MAPK pathways	[110]
Crucian carp	Pancreatin	<1500 Da	IEC-6 cells and DSS-induced colitis mice	0, 50, 100, or 150 *μ*g/mL for 20 h and 50 mg/kg BW for 15 days	Peptides reduced IL-1*β*, IL-6, and TNF-*α* levels via inhibiting the NF-*κ*B pathway	[106]
Soy bean	*B. subtilis* BS12	<3000 Da	Intestinal porcine epithelial cells-J2	50 *μ*g/mL for 2 h	Soy peptides reduced the expression of IL-6, IL-1*β*, and IL-8 and decreased *Escherichia coli* K88-induced inflammation	[4]
Corn	Alcalase and pancreatin	—	TNF-*α*-induced Caco-2 cells and colitis mice	500, 1500, and 2500 *μ*g/mL for 6 h and 100 and 300 mg/kg/d for 14 days	Peptides reduced inflammation via inhibiting IL-8 secretion and iNOS and COX-2 expression and downregulating TNF-*α* and IL-6 expression	[109]

DSS: dextran sodium sulfate; ERK: extracellular signal-regulated kinases; IL: interleukin; TNF-*α*: tumor necrosis factor alpha; MAPK: mitogen-activated protein kinase; NF-*κ*B: nuclear factor-*κ*B; JNK: c-Jun N-terminal kinase.

## Data Availability

No data were used to support this review article.
